# Associations Between the Gut Microbiota and Physical Activity, Sedentary Behaviour and Physical Function in Community‐Dwelling Older Adults

**DOI:** 10.1155/jare/8981398

**Published:** 2026-04-10

**Authors:** Catarina Ramos, Kirsty Hunter, Gemma E. Walton, Anya Whitham, Nicola Camp, Carlos Poveda, Glenn R. Gibson, John Hough, Daniele Magistro

**Affiliations:** ^1^ Department of Sport Science, Sport, Health and Performance Enhancement (SHAPE) Research Centre, Nottingham Trent University, Nottingham, UK, ntu.ac.uk; ^2^ Reynolds Contamination Control, Lincoln, UK; ^3^ Department of Food and Nutritional Sciences, The University of Reading, Whiteknights, Reading, UK, reading.ac.uk

**Keywords:** ageing, gut microbiome, healthy ageing, older adults, physical activity: exercise

## Abstract

Gut microbiota (GM) plays a crucial role in maintaining health through metabolic, endocrine and immune functions. With ageing, shifts in GM composition, characterised by increased pathogenic and decreased health‐promoting bacteria, contribute to dysbiosis, which is linked to several age‐related diseases. Given the global trend of increasing sedentary behaviour (SB) and declining physical activity (PA) among older adults, this study aims to explore the relationships between GM and two critical indicators of healthy ageing, movement behaviours and physical function. Cross‐sectional study assesses the GM composition, PA levels and physical function of 101 healthy, community‐dwelling older adults aged 65–85 years. Participants undertook anthropometric measures and functional tests, wore an accelerometer for 7 days and provided a faecal sample which was analysed using 16s rRNA sequencing. All the results were adjusted for key covariates such as diet, age and activity levels. Key findings include positive associations of *Prevotella copri* with moderate‐to‐vigorous PA, physical function and negative associations with SB, while *Roseburia* species were linked to better mobility and strength measures. Conversely, potentially pathogenic taxa like *Bilophila wadsworthia* and *Eggerthella* were negatively associated with PA and handgrip strength, underscoring their possible detrimental roles in muscle function and healthy ageing. This cross‐sectional study highlights the associations between GM, PA, physical function and healthy ageing in older adults. These findings emphasise the potential for leveraging GM and PA interactions to develop nonpharmacological strategies for promoting healthy ageing, warranting further research through interventional and longitudinal studies.

## 1. Introduction

The world population is not only ageing at an unprecedented rate but also becoming more inactive, with many older adults spending extended periods of time sitting or undertaking sedentary behaviours (SBs). To illustrate, in 2022, the global population of individuals aged over 65 years was 771 million, with projections indicating an increase to 994 million by 2030 and 1.6 billion by 2050 [1]. Data also show that approximately one in four adults fail to meet the recommended levels of physical activity (PA) [2] and that older adults spend, on average, around 10 h in SB per day [3]. SB is associated with higher risk of mortality for all causes and from cardiovascular diseases [4]. Thus, increases in PA and reductions in SB are important for the promotion of healthy ageing, which is defined as the process of developing and maintaining the functional ability that enables wellbeing in older age including the person’s ability to meet their basic needs; be mobile; make their own decisions; build and maintain relationships and contribute to society [5].

Gut microbiota (GM) are the microorganisms (bacteria, archaea and eukarya) that colonise the gastrointestinal tract. Once considered merely facilitators of digestion, these microbes are now recognised as essential regulators of host health. They achieve this through secretion of bioactive metabolites that act locally and systemically, influencing virtually all organs and contributing to metabolic, endocrine and immune functions. The GM is becoming increasingly recognised as a key factor that influences health and disease across the lifespan. This relationship between host health and the GM composition becomes increasingly important as we age because there is a shift in the GM composition that happens together with the ageing process, characterised by an increase in the abundance of potentially pathogenic bacteria, such as those from the Proteobacteria phylum and a decrease in health‐related bacteria, such as *Bifidobacterium, Lactobacillus* and other short‐chain fatty acid (SCFA)‐producing taxa [6]. This creates an imbalance in the GM that is sometimes termed dysbiosis, and this imbalance has been associated with several age‐related diseases and conditions [7]. Strategies to promote healthy ageing ideally slow the rate of dysbiosis, and thus, its contribution to ill health.

One potential approach to mitigating age‐related gut dysbiosis is through PA and its interactions with the GM [8]. In addition to PA, another critical factor associated with healthy ageing is physical function (PF), which is intrinsically linked to PA. PF is defined as the ability to perform both basic and instrumental activities of daily living, and the ability of older adults to reside and engage in the community [9], such as bathing, eating and overall mobility. Previous studies have demonstrated that lower PA levels are associated with diminished PF [10].

Previous research has shown that PA and structured exercise are associated with improvements in the gut microbial composition of older adults [8, 11, 12]. In fact, these studies reported that older adults who were more active had a higher abundance of health‐related bacteria and lower abundance of potential pathogens. However, while the relationship between PA and the GM has been increasingly explored, studies investigating the effects of SB on GM composition remain scarce. Given that older adults typically spend extended periods in sedentary activities, understanding how prolonged SB influences the gut microbiome is critical. Furthermore, little is known about how different elements of PF, such as mobility, upper and lower limb strength and balance, are related to GM composition in older adults. Understanding these associations may also inform targeted interventions, such as microbiome‐modulating strategies or PA prescriptions, to promote healthy ageing and prevent functional decline. Therefore, the aim of this study is to assess if there are any relationships between the GM and PA, SB and PF in community‐dwelling older adults.

## 2. Methods

### 2.1. Ethics Statement and Study Registration

This study was approved by the Nottingham Trent University Research Ethics Committee (application No. 690). Participants provided both electronic and written informed consent before enrolling in the study. They were given comprehensive written information about the study, had the opportunity to ask questions and were informed that they could withdraw at any time without providing a reason. The study was conducted in accordance with the Declaration of Helsinki.

This study did not have a preregistered protocol in a public registry. The observational nature of the cross‐sectional study design did not require prospective registration in a clinical trials database; however, the study protocol is available upon request from the corresponding author.

### 2.2. Study Design and Participants

This observational, cross‐sectional study examined the GM composition and PA levels of 101 healthy, nonsmoking older adults aged 65–85 years, living independently in the United Kingdom. Participants were recruited from the local community via poster advertisements, community centres and word‐of‐mouth referrals. Participants avoided caffeine, alcohol and strenuous activity before the study visit and fasted for at least 10 h.

The planned sample size of 101 participants was based on feasibility considerations (anticipated recruitment capacity within the study period) and informed by previous studies in similar populations. With *n* = 101, a two‐sided test (*α* = 0.05) provides 80% power to detect a correlation of approximately *r* = 0.28 (and 90% power for *r* ≈ 0.32), indicating the study is powered to detect moderate associations but may be underpowered for small effects. Given the large number of taxa tested and false discovery rate correction, power to detect small taxa‐specific associations is likely reduced and findings should be interpreted accordingly.

### 2.3. Inclusion and Exclusion Criteria

Inclusion: (1) 65–85 years old; (2) community‐dwelling; (3) fully vaccinated against COVID‐19; (4) BMI between 18.5 and 35 kg/m^2^.

Exclusion: (1) Routine NSAID use; (2) recent antibiotics; (3) cancer; (4) chronic kidney disease; (5) inflammatory bowel disease; (6) autoimmune diseases; (7) gastrointestinal diseases; (8) smoking; (9) routine pre/probiotic use.

### 2.4. Health Questionnaire and Assessment of Food Intake

Participants completed a health questionnaire and a food frequency questionnaire (FFQ) [13] to estimate nutrient intake. FFQ data were processed using the FFQ EPIC Tool for Analysis (FETA) to identify nutrient extremes and compare cohort intakes with UK averages. This was also used as covariates in the statistical analysis.

### 2.5. Resting Heart Rate, Blood Pressure (BP) and Body Composition

Measurements were collected while fasting. BP was measured twice using an automatic device, and the average was calculated. Height, weight, waist and hip circumference were measured, and BMI and waist‐to‐hip ratio were calculated.

### 2.6. Functional Tests and Cardiorespiratory Fitness Test

The sit to stand test was performed to assess lower‐body strength [14]. Participants were asked to sit on a chair and to fully stand up and sit back down as many times as possible in 30 s, whilst maintaining a proper technique.

The timed up and go (TUG) test was performed to assess mobility [14]. Briefly, participants were asked to sit on a chair and then stand up and walk for 3 m, turn around and sit back on the chair. The test was repeated three times and the best time (shortest amount of time it took to walk back and forth) was used.

The handgrip test was used to assess upper‐body strength. Participants were instructed to squeeze a hydraulic hand dynamometer (JAMAR hand dynamometer) as hard as possible for 5 s. Each hand was measured three times, with the best value for each hand selected. The values for the left and right arms were then averaged.

The 6‐min walking test (6MWT) is a submaximal test used to assess cardiorespiratory fitness. BP, heart rate and the rate of perceived exertion (RPE) [15] were recorded at the end of the test to evaluate the cardiovascular system’s response to exercise.

### 2.7. Assessment of PA, Steps/Day and SB

PA levels and SB were assessed over seven consecutive days using the triaxial accelerometer Actigraph wGT3X‐BT (Actigraph, Pensacola, Florida, USA), worn on the waist. This 1‐week assessment period was chosen to account for variations in activity levels between weekdays and weekends. Data on PA and SB were analysed using the Actilife Software (version 6.13.4). The accelerometer included an inclinometer, enabling the measurement of time spent sitting, lying down and standing.

Participants were advised that the device was not waterproof and therefore to remove it before engaging in any water‐based activities. Participants were asked to provide an exercise diary that stated which water‐based activity they participated in, for how long and any other information that they might have such as metres swam, for example. Additionally, some participants took part in cycling activities and if the accelerometer was placed on the waist, these might not have recorded properly, therefore participants were also asked to provide an exercise diary stating the time spent and distance cycled. The time spent exercising for the above activities was then included in the calculation of PA.

Participants were also told that if the device caused any discomfort during sleep that they could take it off during that period since, in this study, daytime activity level was the only variable of interest.

For data processing and analysis, the device was set to collect units of gravity (1 g = 9.82 m/s^2^) at a sample rate of 100 Hz per second. Data were downloaded and processed using the normal filter option into 30‐s epochs. The Choi algorithm was used to validate wear time (non–wear time was defined as 90 consecutive minutes of 0 counts) with the following parameters: minimum wear time per day of 420 min, minimum of 3 weekdays and 1 weekend day. All non–wear times were excluded from the analysis. The average wear time was 5.1 ± 0.8 days. Of the 101 participants enrolled, 98% met the minimum wear time criteria. Counts per minute (CPM) was used to define PA intensities, with the Freedson adult VM3 (2011) cut points as follows: light (0–2690 CPM), moderate (2691–6166 CPM), vigorous (6167–9642 CPM) and very vigorous (> 9643 CPM). SB was defined as < 100 CPM.

### 2.8. Faecal Sample Collection and Analysis

Participants were given commercial containers (EZ1 Pots with lids, EW Gregory Ltd) to collect faecal samples. These samples were aliquoted and stored at −80°C within 2 h of collection. Microbial DNA was then isolated and extracted from 250 mg of faeces using the QIAamp PowerFecal Pro DNA Kit (QIAGEN, Hilden, Germany), following the manufacturer’s instructions. The final product was 20 μL of purified DNA, with concentrations ranging from 10 to 50 ng/μL per sample. The extracted DNA was stored at −20°C until sequencing.

Faecal DNA samples were shipped to an external company (Eurofins Genomics, Constance, Germany) and sequenced using Next Generation Sequencing. The V3‐V4 (fwd: TACGGGAGGCAGCAG and rev: CCAGGGTATCTAATC) hypervariable regions of the 16sRNA were amplified using Illumina miSeq (PE300 mode). The raw sequencing data were processed using Cutadapt software [16]. The sequences were then demultiplexed, merged and filtered. All reads with ambiguous bases were removed, and the chimeric reads were identified and removed based on the UCHIME algorithm [17]. The high‐quality reads were then processed using the minimum entropy decomposition method, which is an efficient OTU‐picking strategy that can identify and filter random noise in the dataset and posit, and allows decomposition of sequence data sets with a single nucleotide resolution outperforming the classical identity‐based clustering algorithms. OTU assignment was performed using the QIIME software package, and the abundances of bacterial taxonomic units were normalised using lineage‐specific copy numbers of the relevant marker genes to improve estimates.

### 2.9. Statistical Analysis

The microbiome analysis was performed in RStudio (version 2024.04.2 Build 764) using MaAsLin2 R package. MaAsLin 2 is a statistical method to assess multivariable associations between the GM, phenotypes and covariates [18]. MaAsLin2 was run with the following parameters: minimum prevalence of 10%, normalisation method as cumulative sum scaling (CSS), no data transformation, the analysis method was NEGBIN, significance was set at 0.05, Benjamini–Hochberg (BH) was chosen as the method to correct for multiple comparisons, and the significance was set at 0.05. All the models were adjusted for diet (carbohydrates, fat and protein), age, sedentary time and moderate to vigorous physical activity (MVPA). In the Results section, all data presented are significant after BH correction (FDR) and only correlations with coefficients equal to or above 0.1 and/or equal to or below −0.1 are presented.

Microbiome profiling and alpha and beta diversity analyses were performed in the MicrobiomeAnalyst platform [19]. Beta diversity indices (Bray–Curtis, Jaccard’s index and Jensen–Shannon divergence) were analysed using PCoA as the ordination method and pairwise PERMANOVA as the statistical method. The *p*‐value cut‐off for alpha and beta diversity analysis was ≤ 0.05.

## 3. Results

### 3.1. Participants’ Characteristics

A total of 101 community‐dwelling older adults aged between 65 and 85 years living in the United Kingdom took part in the study, and their main characteristics are presented in Tables 1 and 2.

**TABLE 1:** Participant’s characteristics.

Characteristics		Total *n* = 101 (presented as mean ± SD)
Age (years)		71.9 ± 5.3

Sex	Females, *n* (% of sample)	63 (62%)
	Males, *n* (% of sample)	38 (38%)

Anthropometric measures	Height (cm)	167.1 ± 7.7
	Weight (kg)	71.4 ± 12.2
	BMI (kg/m^2^)	25.5 ± 3.4
	Waist circumference (cm)	87.0 ± 10.4
	Hip circumference (cm)	102.1 ± 9.5
	Waist‐to‐hip ratio	0.9 ± 0.1

**TABLE 2:** Cardiovascular, physiological, functional, metabolic and dietary characteristics of the participants included.

Characteristics		Total *n* = 101 (presented as mean ± SD)
BP (mmHg)	Systolic	146 ± 22
	Diastolic	86 ± 11

Resting HR (bpm)		65 ± 11

Blood biomarkers	Glucose (mg/dL)	101.5 ± 13.2
	HDL (mg/dL)	54.8 ± 12.8
	Triglycerides (mg/dL)	109.2 ± 41.0
	LDL (mg/dL)	137.9 ± 39.4
	Total cholesterol (mg/dL)	214.6 ± 4.3

6MWT	Total distance walked (m)	530.8 ± 79.7
	Walking speed (m/min)	88.5 ± 13.3
	RPE	11 ± 2

Estimated VO2max (mL/kg/min)		28.47 ± 6.9

Physical activity	Sedentary time (min)	4927.3 ± 1254.1
	Light PA (min), (%)	8347 ± 1558.1
	Moderate PA (min), (%)	436.7 ± 252.4
	Vigorous PA (min), (%)	21.3 ± 45.1
	Very vigorous (min), (%)	2.8 ± 45.1
	Total MVPA (min)	500.3 ± 320.9
	Average steps/day	7418.2 ± 3201.2

Diet	Carb (g, % of energy)	184.5 ± 62.9 (45.7%)
	Protein (g, % of energy)	71.7 ± 20.5 (18.1%)
	Fat (g, % of energy)	63.9 ± 22.8 (35.5%)

### 3.2. GM Profiling

With regards to phyla, 70% of the identified taxa belonged to Firmicutes, 18% to Bacteroidetes, 7% to Actinobacteria, 3% to Proteobacteria and less than 1% to Tenericutes Of the top 10 most abundant genera, 23.5% belonged to *Faecalibacterium*, 5.29% to *Coprococcus*, 5.38% to *Bacteroides*, 4.55% to *Ruminococcus*, 3.85% to *Collinsella*, 3.46% to *Dorea*, 3.03% to *Roseburia*, 2.55% to *Alistipes* and 2.55% to *Prevotella* (Supporting Figure 1).

**FIGURE 1:**
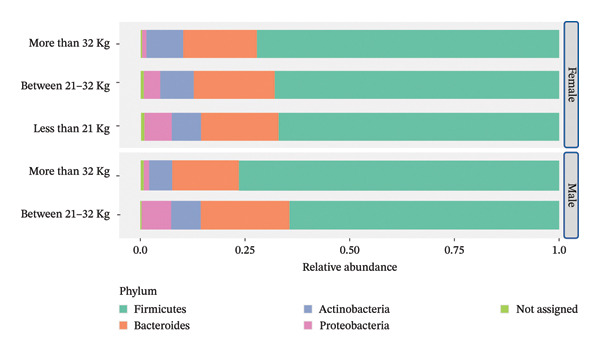
Stacked bar plot showing the relative abundance of taxa grouped by gender and by handgrip strength.

When participants were grouped by handgrip strength and then by gender, the results showed that the relative abundance of Proteobacteria was higher in the groups with the lowest handgrip strength when compared to those who had higher grip strength (Figure 1).

Furthermore, Beta‐diversity assessed using Bray–Curtis index, Jensen–Shannon divergence and Jaccard index all revealed significant differences in the GM diversity among the three grip strength groups (PERMANOVA, *R* ^2^ = 0.029, *p* = 0.024, *R* ^2^ = 0.041, *p* 0.007, *R* ^2^ = 0.025, *p* = 0.043, respectively). This suggests that the GM composition of older adults with different handgrip strength is distinct. More specifically, pairwise comparisons indicated that the GM composition of the above 32 kg group was significantly different from the 21–32 kg group (FDR = 0.003 for Bray–Curtis index and Jensen–Shannon divergence and FDR = 0.06 for Jaccard index) (Supporting Figure 2).

There were no significant differences in all the other measures of alpha and beta diversity for the other variables.

### 3.3. Associations Between Gut Microbial Taxa and PA

All of the data regarding the MaAslin correlation coefficients for all taxa are presented in the supporting file (available here).

### 3.4. Light Physical Activity (LPA)

Overall, 48 taxa were significantly associated with LPA in healthy older adults (Figures 2(a) and 2(b)). Among these, strong negative correlations with LPA were observed for *Senegalimassilia anaerobia, Angelakisella massiliensis, caproiciproducens galactitolivorans, Collinsella bouchesdurhonensis, Coprooccus* sp. ART55.1*, Dorea* sp. Marseille P3386*, Intestiminonas butyriciproducens, Negativibacillus massiliensis, Olsenella,* several members of the Parabacteroides genus including *P. distasonis* and *P. johnsonii, Ruminococcus clarus, Paraprevotella clara* and *Turicibacter sanguinis.* In contrast*,* strong positive correlations were found with two *Bacteroides* species (*B. finegoldii* and *B. fragilis), Barnesiella intestihominis, Blautia stercoris,* three *Clostridium* species (*C. cellulovorans, C. polysaccharolyticum* and *C.* AN.AS8*)*, *Coprococcus* sp.*, Coprococcus eutactus, Eggerthella* sp., *Hungateiclostridium clariflavum, Kineothrix* sp., *Massiliprevotella massiliensis, Ruminococcus champanellensis* and *Valittalea pronyensis.*

FIGURE 2(a) Heatmap showing associations between taxa and movement behaviours. SB: Sedentary behaviour; LPA: light physical activity; MPA: moderate physical activity; VPA: vigorous physical activity. All the associations presented were FDR‐corrected and are significant at *p* ≤ 0.05. (b) Heatmap showing associations between taxa and movement behaviours. SB: sedentary behaviour; LPA: light physical activity; MPA: moderate physical activity; VPA: vigorous physical activity. All the associations presented were FDR‐corrected and are significant at *p* < 0.05.

**Figure:**
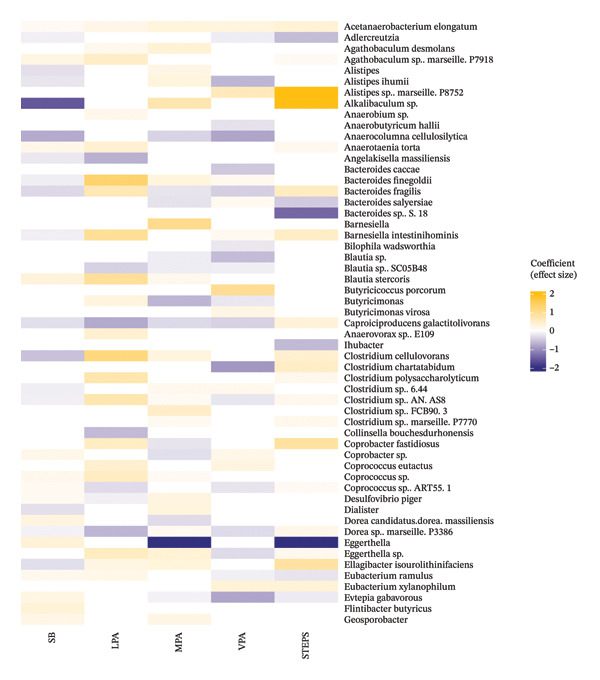
(a)

**Figure:**
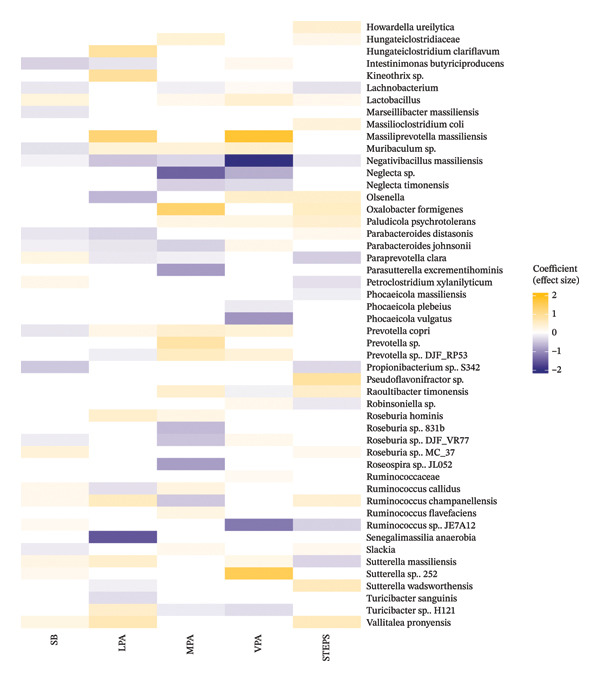
(b)

### 3.5. Moderate to Vigorous PA

We identified 57 taxa that were significantly associated with moderate physical activity (MPA) in community‐dwelling older adults (Figures 2(a) and 2(b)). *Eggerthella* genus showed a very strong negative correlation with MPA. Other taxa negatively associated with MPA included *Evtepia* g*abavorous, Butyricimonas,* three *Blautia* species (Unclassified *Blautia, B. stercoris* and *B.* SC05B48), two *Bacteroides* species (*B. fragilis* and *B. slayersiae) Neglecta* sp.*, Parasuterrella excrementihomins, Roseospira* JL052, two *Roseburia* species (*R.* 831b and R.DJF_VR77)*, Ruminococcocus champanellensis, Parabcteroides johnsonii* and *Paraprevotella clara.* Conversely, *Alkalibaculum* sp., *Barnesiella, Oxalobacter formigenes* and *Prevotella* sp. were all strongly positively associated with MPA. Other taxa positively associated with MPA included the genus *Alistipes* and its species—*A. ihumii,* the family Hungateiclostridiaceae, members of the *Prevotella* genus (*P. copri* and *P.* DJF_RP53), *Raoultibacter timonensis, Roseburia hominis*, two *Ruminococcus* species (*R. Callidus* and *R. flavefaciens*) and the genus *Slackia.*

### 3.6. Vigorous Physical Activity (VPA)

In relation to VPA, we identified 52 taxa significantly associated with it in community‐dwelling older adults (Figures 2(a) and 2(b)). Strong negative associations with VPA were observed for *Negativibaccillus massiliensis, Phocaeicola vulgatus, Clostridium chartatabidum* and *Ruminococcus* JE7A12. Other taxa negatively associated with VPA included *Anaerostipes ihumii, Anaeroclumna cellulosilytica,* two *Bacteroides* species (*B. fragilis* and *B. caccae), Blautia* sp., *Bilophila wadsworthia, Eggerthella* sp. and *Evtepia gabavorous.* The opposite was found for *Butyricoccos porcorum, Masilliprevotella massiliensis* and *Sutterella* sp. 252 species, which were found to be very strongly correlated with VPA. Additional positive associations were found for *Butyricimonas virosa, Coprobacter* sp., *Coprococcus eutactus, Eubacterium xylanophilum, Lactobacillus, Muribaculum, Olsenella, Paludicola psychrotolerans* and two *Prevotella* species (*P. copri* and *P. DJF_RP53*).

### 3.7. Step Count

Fifty taxa were significantly associated with the number of steps per day in the older adults (Figures 2(a) and 2(b)). Among these, *Eggerthella*, *Bacteroides* sp. S18 and *Ihubacter* showed a strong negative association with the number of steps per day. Alongside these, *Adlercreutzia, Bacteroides salyersiae, Eubacterium ramulus, Evtepia gabavorous, Lachnobcterium, Paraprevotella clara, Petroclostridium xylanilyticum* and *Sutterella massiliensis* were also negatively associated with steps/day. Conversely, an *Alistipes* species, *Alkalibaculum* sp., *Coprobacter fastidiosus, Ellagibacter isourolithinifaciens* and *Pseudoflavonifractor* sp. showed strong positive associations with the number of steps per day. Other positively associated taxa included *Barnesiella intestinihominis,* two *Clostridium* species (*C. cellulovorans* and *C. chartatabidum), Eubacterium xylanophilum, Howardella ureilytica, Olsenella, Oxalobacter formigenes, Paludicola psychrotolerans, Raoultibacter timonensis, Ruminoccoccus champanellensis, Sutterella wadsworthesis* and *Vallitalea pronyensis.*

### 3.8. SB

Fifty‐two taxa were significantly associated with time spent in SB amongst community‐dwelling older adults (Figures 2(a) and 2(b)). The taxa most strongly negatively associated with SB were *Alkalibaculum* sp., *Anaercolumna cellulosilytica, Clostridium cellulovorans* and *Propionibacterium* sp. Other taxa inversely associated with SB included *Alistipes, Alistipes ihumii, Angelakisella massiliensis,* two *Bacteroides* species (*B. fragilis* and *B. finegoldii*)*, Barnesiella intestinihominis, Dialister, Ellgaibacter isourolithinifaciens, Intestinimonas butyriciproducens, Lachnobacterium Muribaculum* sp., *Slackia* and two *Parabacteroides* species (*P. distasonis* and *P. johnsonii*)*.* Conversely, *Anaerotaenia torta, Coprobacter* sp., *Coprococcus* sp., *Dseulfovibrio piger, Eggerthella, Evtepia gabavorous, Flintibacter butyricus, Paraprevotella clara, Petroclostridium xylanilyticum,* two *Sutterella* species (*S. massiliensis* and *S.* sp. 252), *Vallitalea pronyensis* and two *Ruminococcus* species (*R. Callidus* and *R. champanellensis*) were positively associated with SB.

### 3.9. Associations Between Gut Microbial Taxa and PF

All of the data regarding the MaAslin correlation coefficients for all taxa are presented in the supporting file (available here).

### 3.10. Handgrip

We identified 52 significant associations between taxa and handgrip strength (measure by handgrip) and 41 associations between taxa and handgrip strength normalised to body weight (%Handgrip) in community‐dwelling older adults (Figures 3(a), 3(b)). Handgrip was strongly negatively associated with *Howardella ureilytica, Caproiciproducens galactitolivorans* and *Pseudoflavonifractor* sp., while *Clostridium TG60.1* and *Bacterodies caccae* were strongly negatively associated with %Handgrip. Other negative correlations between taxa and Handgrip included *Butyricimonas virosa, Clostridium polysaccharolyticum, Eggerthella,* Hungateiclostridiaceae and *Petroclostriidum xylanilyticum.* Additionally, *Angelakisella massiliensis, Alistipes ihumii*, Bacteroidales*, Ellagibacter isourolithinifaciens, Blautia* sp. SC05B48, *Clostridium chartatabidum* and *Massilioclostridium coli* were found to be negatively associated with both Handgrip and %Handgrip. *Adlercreutzia equolifaciens, Blautia* sp., *Ihubacter,* Parabacteroides and two *Sutterella* species (*S. massiliensis* and *S. wadsworthensis*) were other taxa negatively associated with %Handgrip.

FIGURE 3(a) Heatmap showing associations between taxa and functional capacity. Handgrip stands for handgrip strength in kg and %Handgrip is the handgrip strength in kg normalised to the participant’s body weight. TUG: Timed up and go. All the associations presented were FDR‐corrected and are significant at *p* < 0.05. (b) Heatmap showing associations between taxa and functional capacity. Handgrip stands for handgrip strength in kg and %Handgrip is the handgrip strength in kg normalised to the participant’s body weight. TUG: Timed up and go. All the associations presented were FDR‐corrected and are significant at *p* < 0.05.

**Figure:**
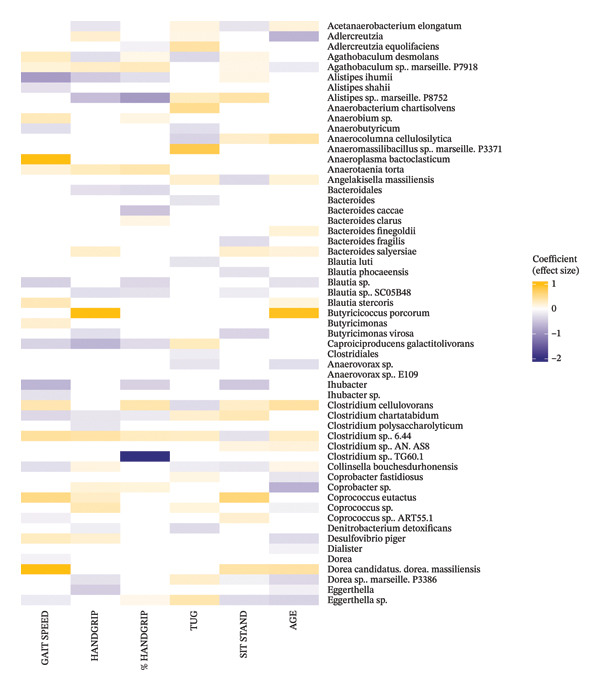
(a)

**Figure:**
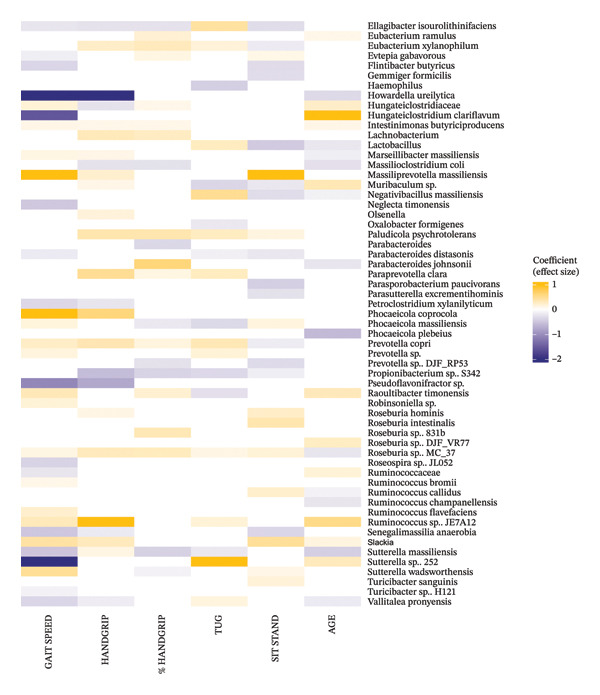
(b)

Regarding positive associations, *Butyriciccocus porcurum, Ruminococcuss* sp. JE7A12, *Phocaeicola coprocola* and *Paraprevotella clara* were strongly associated with Handgrip strength, while *Parabcteroides johnsonii* was found to be strongly associated with %Handgrip. Other positive associations between taxa and Handgrip included *Bacteroides salyersiae, Clostridium* sp. 6.44*, Coprococcus* sp., *Coprococcus eutactus, Eubacterium xylanophilum, Olsenella, Paludicola psychrotolerans, Prevotella copri, Roseburia hominis* and *Slackia*. For %Handgrip, several taxa such as *Anaerobium* sp.*, Bacteroides clarus, Clostridium cellulovorans,* two *Eubacterium* species (*E. ramulus* and *E. xylanophilum*), *Paludicola psychrotolerans, Prevotella copri* and two *Roseburia* species were positively associated with this variable.

### 3.11. TUG

Forty‐seven taxa were associated with the TUG test performance in the community‐dwelling older adults (Figures 3(a), 3(b)). Negative associations with TUG were observed for *Anaerobutyricum, Anaeroclumna cellulosilityica, Anerovorax* sp., Clostridiales, *Clostridium cellulovorans, Denitrobacterium detoxificans, Hameophilus, Muribaculum* sp*, Phocaeicola massiliensis* and *Raoultibacter timonensis*. Conversely, *Sutterella* sp. 252 and a *Anaeromassilibacillus* species showed a very strong positive association with TUG. Other taxa positively associated with TUG included *Adlercreutzia equolifaciens, Anaerobacterium chartisolvens, Clostridium chartatabidum, Ellagibacter isourolithinifaciens, Eggerthella* sp.*, Lactobacillus, Negativibacillus massiliensis, Paraprevotella clara* and *Prevotella copri*.

### 3.12. Sit to Stand

Regarding the sit to stand, we found 48 taxa associated with the older adults’ performance in this test (Figures 3(a), 3(b)). Negative associations with the sit to stand performance were observed for *Angelakisella massiliensis, Bacteroides fragilis, Blautia phocaeensis, Butyricimonas virosa, Ihubacter, Eggerthella* sp.*, Ellagibacter isourolithinifaciens, Flintibacter butyricus, Gemmiger formicilis, Lactobacillus, Parasporobacterium paucivorans,* two *Prevotella* species (*P. copri* and *Prevotella* sp. DJF_RP53) and *Senegalimassilia anaerobia*. Conversely, *Massiliprevotella massiliensis* and *Coprococcus eutactus* were strongly positively associated with sit to stand test. Other taxa positively associated with this test included *Clostridium chartatabidum, Dorea massiliensis, Roseburia intestinalis, Roseburia hominis, Ruminococcus Callidus* and *Slackia.*

### 3.13. Gait Speed

Fifty‐six taxa were associated with gait speed in the healthy community‐dwelling older adults (Figures 3(a), 3(b)). Strong negative associations with gait speed were observed for *Alistipes ihumii, Ihubacter, Howardella ureilytica, Hungateiclostridium clariflavum, Pseudoflavonifractor* sp. and *Sutterella* sp. 252*.* Other taxa negatively associated with gait speed included *Blautia* sp.*, Neglecta timonensis, Petroclostridium xylanilitycum, Senegalissamilia anaerobia, Suterella massiliensis* and *Vallitalea pronyensis*. Conversely, strong positive associations with gait speed were found for *Aneroplasma bactoclasticum, Dorea massiliensis, Massiliprevotella massiliensis* and *Phocaeicola coprocola*. Additional positive associations included *Anaerobium so, Blautia stercoris, Butyricimonas, Clostridium cellulovorans, Clostridium* sp. 6.44*, Coprococcus eutactus, Raoultibacter timonensis, Slackia* and *Suterella wadsworthensis*.

## 4. Discussion

The aim of this study was to investigate associations between members of the GM, movement behaviours (PA and SB) and PF in community‐dwelling older adults. A multivariable association test was applied to explore the relationships between gut microbial taxa and measures of PA, including light (LPA), moderate (MPA) and vigorous (VPA) intensity, as well as step count, SB and various domains of PF. These domains included functional ability (gait speed), upper‐body strength (handgrip and %handgrip), lower‐body strength (sit‐to‐stand), mobility and mobility (TUG). Dietary components, age and PA were included as covariates in the analysis. Several taxa were identified as being significantly associated with movement behaviours and PF in healthy older adults.

*Prevotella* species (*P. copri* and *Prevotella* DJF_RP53) were shown to be consistently positively associated with all types of PA and *Prevotella* sp. was strongly associated with just MPA. These species were also positively associated with PF, mainly gait speed, handgrip, %handgrip and TUG. Additionally, *P. copri* was negatively associated with SB. The same result was found by Baldanzi and colleagues [20], in which *P. copri* was also negatively associated with SB. Additionally, previous studies have shown that the Prevotellaceae family was positively associated with arm and leg muscle mass in older adults [21]. Moreover, *Prevotella* genus and *P. copri* abundance were lower in sarcopenic patients (25). Interestingly, Wang and colleagues [22] found that centenarians had higher abundance of this genus. *P. copri* has additionally been negatively associated with fatty liver, constipation, atherosclerosis, inflammatory bowel disease, irritable bowel syndrome and geriatric depression scale in an aged cohort [14]. Altogether, these findings suggest that *Prevotella* genus, and more specifically, *P. copri* might be a health‐related species in older adults and that it might be associated with healthy ageing, PF and PA in older adults.

*Roseburia hominis* was positively associated with LPA, MPA, handgrip strength and sit to stand test in older adults. *Roseburia intestinalis* was also positively associated with performance on the sit to stand test. Recent research has shown that both species might be associated with health. For instance, *Roseburia hominis* was shown to be positively associated with general health and plant protein intake and negatively associated with glycaemic load, caloric intake [15], insulin resistance, blood triglycerides [23], Type 2 diabetes, negative functional independence and negative mini mental state examination in an aged cohort [14]. *Roseburia intestinalis* was also negatively associated with Type 2 diabetes, fatty liver, diarrhoea, constipation, colorectal cancer and negative functional independence in an aged cohort [14]. *R. intestinalis* is an abundant butyrate‐producer in the gut [16] and has been shown to play a role in the regulation of the immune system, cytokine release and gut barrier homeostasis, via butyrate and flagellin action [17]. This species has been shown to be typically associated with health and healthy individuals [18]. Taken together, these findings suggest that these two species are likely associated with healthy ageing, PF and mobility at an older age.

*Bilophila wadsworthia*, a common gut pathogen, was negatively associated with VPA. *B. wadsworthia* uses taurine to produce hydrogen sulphide, a known metabolite that negatively affects the intestinal environment [24]. Additionally, it was shown to be able to secrete pro‐inflammatory cytokines, promote gut barrier permeability, systemic inflammation and bile acid dysmetabolism and promote adverse changes in the gut microbiome functionality [19]. These results suggest that older adults who engage in higher amounts of VPA have lower abundances of this species, and this reduced abundance might help promote healthy ageing.

*Eggerthella* genus was strongly negatively associated with MVPA and number of steps per day and also with handgrip strength. Conversely, it was positively associated with SB. These results align with previous studies since this genus has been shown to be more abundant in patients with sarcopenia and negatively correlated with muscle mass index [25, 26]. Altogether, this suggests that this genus may be detrimental to muscle mass in older adults via the gut–muscle axis. However, there are some studies with contrasting findings such as [27] and these contrasting findings may be due to different population characteristics, different statistical analyses or even different 16s rRNA hypervariable regions chosen.

*Paludicola psychrotolerans* was positively associated with PA (MPA, VPA and number of steps) and with several domains of PF such as handgrip, TUG and sit to stand. Little is known about this species in the literature, but based on our results, it seems to be associated with health, movement, PA, PF and therefore healthy ageing.

*Slackia* was positively associated with MVPA, number of steps per day and several domains of PF such as gait speed, handgrip strength and sit to stand test performance. These results align with a previous study that showed that this genus was less abundant in sarcopenic patients [27], consequently suggesting a health benefit and a relationship between the GM and muscle mass (gut–muscle axis).

*Alistipes* was positively associated with MVPA and negatively associated with SB. The same trend was found for *Alkalibaculum* sp. There are several contrasting results in the literature about whether *Alistipes* is a health‐related or pathogenic bacteria [28]. Interestingly, a study in older women found that this genus was more abundant in women who lived longer, and it was suggested that it might be involved in a pathway related to longevity [29]. Regarding *Alkalibaculum* sp., there is not much known about this taxa. Nonetheless, our results suggest that these taxa might be associated with healthy ageing and PF in community‐dwelling older adults.

Bacteroidales order was negatively associated with handgrip (handgrip strength and %handgrip), whereas *Bacteroides* genus was negatively associated with TUG. Additionally, *Bacteroides caccae* was negatively associated with VPA and %handgrip, and *Bacteroides* sp. S18 was negatively associated with the number of steps per day. On the contrary, *Bacteroides clarus* was positively associated with %handgrip, and *Bacteroides finegoldii* was positively associated with three PA intensities (LPA, MPA and VPA) and negatively associated with SB. These results suggest that Bacteroidales, *Bacteroides*, *Bacteroides caccae* and *Bacteroides* sp. 18 abundances seem to be associated with poorer handgrip and PF at an older age. Previous studies have shown that *Bacteroides* were negatively associated with walking or biking [30] and people with low muscle mass index had higher abundance of this genus [31]. Furthermore, *B. caccae* has been linked to IBD [32]. On the other hand, *Bacteroides clarus* and *Bacteroides finegoldii* seem to be health‐related taxa since they are positively associated with PA, handgrip and negatively associated with SB. Little is known about *B. finegoldii* physiological effects/associations and the same goes for *B. clarus.* However, previous studies have shown that *B.* clarus was enriched in controls compared to colorectal cancer patients [33], thus suggesting that it might be associated with overall health and healthy ageing.

*Barnesiella* was strongly associated with MVPA and *Barnesiella intestinihomins* was positively associated with LPA, VPA and the number of steps per day and negatively associated with SB. While a previous study in older adults showed that an unclassified species of this genus was less abundant in older adults with low muscle mass [34], the potential positive influence of *B. intestinihominis* was demonstrated with negative associations with triglycerides, insulin resistance [23], Type 2 diabetes, negative functional independence, negative mini mental state score and geriatric depression score in an aged cohort [14]. Altogether, these results suggest that these taxa might be implicated in PF, muscle function and healthy ageing.

*Butyriciccocus procurum* showed very strong associations with VPA and handgrip strength. *B porcorum* is a butyrate producer [35] and, according to our results, it might be involved in the process of healthy ageing due to its associations with VPA and handgrip. Not much is known about this species in the literature; therefore, more research is needed to properly understand its effects/implications *n* healthy ageing and PF.

*Ihubacter* and *Ihubacter* sp. were negatively associated with the number of steps per day, and PF. *Ihubacter* is a key microbe involved in the production of trimethylamine N‐oxide (TMAO) [36], which is a metabolite that has been associated with cancer, atherosclerosis, chronic kidney disease and inflammation [37]. These results suggest that this microbe may be negative to host health and lower abundances of it might be beneficial at an older age.

*Clostridium cellulovorans* was negatively associated with SB and positively associated with LPA, MPA, steps per day and PF; the same was true for *Clostridium* 6.44 and *Clostridium* AN AS8*.* In addition, *Clostridium* FCB90 and *Clostridium* Marseille P7770 also showed positive associations with MPA in community‐dwelling older adults. There is limited research on the effects of these taxa on health/disease, however, it is known that *C. cellulovorans* is a butyrate producer [38], which is a metabolite beneficial for health. Based on these results, it seems that these species belonging to the *Clostridium* genus seem to be associated with health, PA, PF and therefore healthy ageing. *Coprococcus eutactus* was positively associated with LPA, VPA, gait speed, handgrip and sit to stand test. These results align with previous research that showed that this species was negatively associated with Type 2 diabetes, kidney stones, IBD, IBS and negative functional independence [14]. Interestingly, it was also found to be negatively associated with SB [20] and chair rise time [39]. Altogether, these results suggest that this species is related to health and healthy ageing due to its positive associations with PA, gait speed and handgrip which are all determinants of healthy ageing.

Another noteworthy finding was that three beta‐diversity indices (Bray–Curtis, Jensen–Shannon and Jaccard) all revealed significant differences in GM composition between the different handgrip strength groups. In particular, pairwise comparisons showed that individuals with handgrip strength above 32 kg had a distinct GM composition compared to those in the 21–32‐kg group. Although beta‐diversity measures do not identify specific microbial taxa, they indicate that the overall community structure differs between groups, suggesting that handgrip strength may be associated with distinct gut microbial profiles in older adults. This is particularly compelling given that handgrip strength is a well‐established biomarker of general health [40], associated with quality of life [41] and reflective of functional independence in older age [42]. These findings suggest that handgrip strength could serve as a useful phenotypic marker when investigating GM‐related health trajectories in ageing. However, further research is needed to better understand the mechanisms underlying this association and to clarify the potential role of handgrip strength in shaping the GM composition of older adults.

This study has several strengths: (1) PA and SB were measured objectively with accelerometers over 7 days, providing reliable data; (2) dietary intake was estimated through a detailed FFQ, enabling key dietary components to serve as covariates in the analysis and (3) strict inclusion and exclusion criteria minimised the influence of diseases, conditions, or medications known to impact the GM, offering a clearer view on the associations between taxa and movement behaviours and PF.

However, some limitations should be noted: (1) the cross‐sectional design limits causal conclusions. (2) The 16S rRNA sequencing technique, while widely used, has limitations, including lower resolution, lack of functional insight and potential biases due to primer and hypervariable region selection. While is a cost‐effective approach to profiling bacterial composition, it has limited discriminatory power at the species level. Whole‐genome sequencing or metagenomics would be required to definitively identify species‐specific mechanisms. (3) The sample population was more physically active and physically fit than the general older adult population, which may limit the generalisability of the findings. This likely reflects a self‐selection bias, as individuals with high levels of SB are less likely to volunteer for studies involving physical assessments. As a result, the sample may underrepresent more sedentary, frail or health‐compromised individuals. (4) Only lower GM were assessed via faecal samples. (5) Accelerometers placed on the waist may not have accurately captured all activities, particularly cycling. (6) Although we adjusted for total carbohydrate, fat and protein intake, dietary factors such as fibre intake, which are known to influence GM composition, were not specifically modelled and may have contributed to residual confounding.

Future research should focus not only on the GM composition, but also in its functionality and more studies should be performed in this population in order to understand the relationships between GM composition, PF and health ageing.

## 5. Conclusion

This study provided insights into the relationship between GM composition, movement behaviours and PF, in community‐dwelling older adults. Specific taxa were positively associated with PF and PA, including several SCFA producers that may play a role in the process of healthy ageing. Other taxa were negatively associated with parameters linked to healthy ageing, potentially indicating a role in poor health in older age.

Identifying associations between specific taxa, movement behaviours and PF in older adults can provide a deeper understanding of the complex interaction between the GM and healthy ageing. These novel results could inform the development of nonpharmacological strategies to promote healthy ageing through PA and GM modulation. However, further interventional and longitudinal studies are needed to clarify the relationship between GM, PF, PA and healthy ageing.

## Funding

This research was supported by internal funding from Nottingham Trent University (redacted for revision).

## Conflicts of Interest

The authors declare no conflicts of interest.

## Supporting Information

Beta‐diversity indexes: PCoA plots of Beta diversity of older adults with different handgrips strength based on: A: Bray–Curtis dissimilarity measure; B: Jensen–Shannon divergence; C: Jaccard index.

MaAsLin2 correlations: MaAslin correlation coefficients for all taxa.

Supporting Figure 1: Area plot showing the top 10 genera in the participants’ gut microbiome.

## Supporting information


**Supporting Information** Additional supporting information can be found online in the Supporting Information section.

## Data Availability

The data that support the findings of this study are available upon request from the corresponding author, Catarina Ramos. The data are not publicly available due to containing information that could compromise the privacy of research participants. The data are pseudonymised and have been uploaded to Zenodo under the DOI: 10.5281/zenodo.12799150 and their access is restricted.
