# Supplementary material for: Associations Between the Gut Microbiota and Physical Activity, Sedentary Behaviour and Physical Function in Community‐Dwelling Older Adults
Source: J Aging Res. 2026 Apr 10;2026:8981398. doi: 10.1155/jare/8981398 (PMC13069175; doi:10.1155/jare/8981398)
Supplement: Supplementary file 1 — Supporting Information Additional supporting information can be found online in the Supporting Information section. [file JARE-2026-8981398-s001.zip › Maaslin correlations.pdf]

MaAsLin Correlations

Feature	SB	LPA	MPA	VPA	STEPS	GAIT SPEED	HAND GRIP	%HAND GRIP	TUG	SIT STAND	AGE
Acetanaerobacterium elongatum	0.10	0.23	0.33	0.31	0.39		-0.20		0.14	-0.22	0.17
Adlercreutzia	-0.13			-0.14	-0.57		0.22		0.12		-0.67
Adlercreutzia equolifaciens								-0.11	0.42		
Agathobaculum desmolans		0.17	0.39			0.29	-0.28	0.11	- 0.34	0.12	
Agathobaculum sp..Marseille.P7918	0.26	0.52			0.11	0.19	0.26	0.32		0.12	-0.17
Alistipes	-0.27		0.24								
Alistipes ihumii	-0.21		0.33	-0.65		-0.87	-0.45	-0.27		0.12	
Alistipes shahii						-0.27					
Alistipes sp..Marseille.P8752				0.65	2.35		-0.57	-0.90	0.30	0.41	
Alkalibaculum sp.	-1.54		0.74		1.96						
Anaerobacterium chartisolvens									0.52		
Anaerobium sp.		0.20				0.32		0.14			
Anaerobutyricum						-0.28			- 0.27		
Anaerobutyricum hallii				-0.26							
Anaerocolumna cellulosilytica	-0.74		-0.38	-0.79					- 0.38	0.25	0.40

Anaeromassilibacillus sp..Marseille.P3371									0.80		
Anaeroplasma bactoclasticum						1.12					
Anaerotaenia torta	0.20	0.43			0.15	0.18	0.30	0.38			
Angelakisella massiliensis	-0.18	-0.68							0.23	-0.32	0.19
Bacteroidales							-0.27	-0.30			
Bacteroides									-0.24		
Bacteroides caccae				-0.48				-0.51			
Bacteroides clarus								0.12			
Bacteroides finegoldii	-0.13	1.36	0.33	0.15							0.19
Bacteroides fragilis	-0.34	0.69	-0.23	-0.42	0.58					-0.29	
Bacteroides salyersiae			-0.24	0.13	-0.46		0.24			0.23	0.17
Bacteroides sp..S.18					-1.40						
Barnesiella			1.06								
Barnesiella intestinihominis	-0.13	0.97		0.13	0.53						
Bilophila wadsworthia				-0.19							
Blautia luti									-0.24		
Blautia phocaeensis										-0.26	
Blautia sp.			-0.14	-0.57		-0.40		-0.34			-0.25
Blautia sp..SC05B48		-0.39	-0.14	-0.14			-0.28	-0.24		-0.15	
Blautia stercoris	0.34	0.94	0.17			0.33					0.17
Butyricicoccus porcorum				0.99			1.18				0.95
Butyricimonas		0.31	-0.65	-0.19		0.22					
Butyricimonas virosa				0.27			-0.28			-0.37	
Caproiciproducens	-0.27	-0.75	-0.32	-0.39	0.36	-0.39	-0.64	-0.30	0.31		

galactitolivorans											
Clostridiales									-0.15		
Anaerovorax sp.									-0.22		-0.28
Anaerovorax sp..E109		0.42									
Ihubacter					-0.60	-0.65		-0.39		-0.48	
Ihubacter sp.						-0.26					
Clostridium cellulovorans	-0.53	1.19	0.32		0.44	0.36		0.38	-0.31	0.25	0.43
Clostridium chartatabidum				-0.92	0.52	-0.34	-0.21	-0.17	0.24	0.34	
Clostridium polysaccharolyticum		0.76			0.18		-0.22				
Clostridium sp..6.44	-0.14		0.16	0.16		0.45	0.40	0.30	0.27	-0.24	0.27
Clostridium sp..AN.AS8	-0.12	0.75	0.10	-0.22	0.17					0.14	0.17
Clostridium sp..FCB90.3			0.50								
Clostridium sp..Marseille.P7770			0.11		0.19						
Clostridium sp..TG60.1								-4.41			
Collinsella bouchesdurhonensis		-0.60				-0.27	0.15		-0.16	-0.18	0.11
Coprobacter fastidiosus		0.55	-0.23		0.89				0.13		-0.22
Coprobacter sp.	0.19		-0.28	0.22			0.19	0.17			-0.67
Coprococcus eutactus		0.45		0.29		0.56	0.26			0.63	
Coprococcus sp.	0.17	0.57	0.10				0.35		0.16		-0.11
Coprococcus sp..ART55.1	0.11	-0.30		-0.23	0.10	-0.13				0.23	
Denitrobacterium detoxificans							-0.14		-0.32		
Desulfovibrio piger	0.11	-0.13	0.30			0.28	0.21				-0.30

Dialister	-0.27		0.33								-0.11
Dorea						-0.10					
Dorea Candidatus.Dorea.massiliensis	0.30		-0.30			1.16				0.41	0.42
Dorea sp..Marseille.P3386	-0.11	-0.65	0.12	-0.31	0.21		-0.26		0.25	-0.11	-0.33
Eggerthella	0.35		-5.30		-5.74		-0.42				-0.13
Eggerthella sp.		0.58	0.39	-0.31	0.16	-0.17		0.10	0.38	-0.31	-0.38
Ellagibacter isourolithinifaciens	-0.28	0.27	0.34		0.86	-0.21	-0.25	-0.24	0.44	-0.30	
Eubacterium ramulus	0.14	0.22		-0.12	-0.23			0.21			0.11
Eubacterium xylanophilum				0.38	0.39		0.25	0.31	0.18	-0.16	
Evtepia gabavorous	0.28		-0.11	-0.79	-0.16	-0.13		0.14		0.11	
Flintibacter butyricus	0.38					-0.35				-0.30	
Gemmiger formicilis										-0.28	
Geosporobacter	0.23		0.24								
Haemophilus									-0.42		
Howardella ureilytica					0.44	-3.79	-4.82				-0.32
Hungateiclostridiaceae			0.39		0.20	0.19	-0.24	0.11			0.26
Hungateiclostridium clariflavum		0.89				-1.56					1.92
Intestinimonas butyriciproducens	-0.39	-0.23		0.15		0.12	0.11	0.10			0.12
Kineothrix sp.		0.93									
Lachnobacterium	-0.19		-0.12	0.10	-0.25		0.32	0.28			
Lactobacillus	0.33		0.18	0.43	0.16				0.30	-0.45	-0.19
Marseillibacter massiliensis	-0.22					0.13	0.11				-0.13

Massilioclostridium coli					0.34		-0.24	-0.25			-0.27
Massiliprevotella massiliensis		1.28		1.81		1.78	0.23			2.27	
Muribaculum sp.	-0.25	0.38	0.36	0.49			0.11		-0.36	-0.19	0.35
Negativibacillus massiliensis	-0.11	-0.50	-0.35	-2.37	-0.20				0.50	-0.30	-0.12
Neglecta sp.			-1.44	-0.71							
Neglecta timonensis			-0.40	-0.31		-0.48					
Olsenella		-0.63		0.48	0.49		0.17				
Oxalobacter formigenes			1.36		0.55				-0.18		
Paludicola psychrotolerans			0.25	0.27	0.44		0.38	0.38	0.29	0.14	
Parabacteroides								-0.34			
Parabacteroides distasonis	-0.20	-0.38			0.16	-0.17			-0.12	-0.19	
Parabacteroides johnsonii	-0.13	-0.21	-0.40	0.19				0.62			-0.21
Paraprevotella clara	0.28	-0.18	-0.12		-0.44		0.50	0.14	0.30		
Parasporobacterium paucivorans										-0.41	
Parasutterella excrementihominis			-0.87							-0.25	
Petroclostridium xylanilyticum	0.20				-0.27	-0.33	-0.22				
Phocaeicola coprocola						1.49	0.61				
Phocaeicola massiliensis					-0.13	0.17		-0.18	-0.32	0.16	
Phocaeicola plebeius				-0.18							-0.60
Phocaeicola vulgatus				-0.94							

Prevotella copri	-0.22	0.22	0.44	0.38		0.26	0.35	0.15	0.34	-0.15	
Prevotella sp.			0.82			0.16			0.19		
Prevotella sp..DJF_RP53		-0.14	0.57	0.36				-0.26		-0.31	
Propionibacterium sp..S342	-0.48				-0.33		-0.57	-0.38	-0.33	-0.14	
Pseudoflavonifractor sp.					0.88	-1.07	-0.77				
Raoultibacter timonensis			0.45	-0.11	0.50	0.33		0.21	-0.26		0.32
Robinsoniella sp.				0.18	-0.18	0.19					
Roseburia hominis		0.48	0.24				0.12			0.26	
Roseburia intestinalis										0.37	
Roseburia sp..831b			-0.59					0.34			
Roseburia sp..DJF_VR77	-0.15		-0.52	0.18							0.28
Roseburia sp..MC_37	0.37				0.14	0.14	0.31	0.30	0.12	0.19	-0.22
Roseospira sp..JL052			-0.85			-0.39					
Ruminococcaceae				0.11		-0.21					0.19
Ruminococcus bromii						0.11					
Ruminococcus callidus	0.19	-0.27	0.30							0.24	-0.10
Ruminococcus champanellensis	0.18	0.60	-0.47		0.41						-0.21
Ruminococcus flavefaciens			0.26			0.24					
Ruminococcus sp..JE7A12	0.12			-1.22	-0.39	0.32	1.96		0.19		0.55
Senegalimassilia anaerobia		-1.57				-0.49	-0.17			-0.35	
Slackia	-0.16		0.19		0.15	0.42	0.32			0.48	0.15
Sutterella massiliensis	0.25	0.48		0.22	-0.36	-0.51	0.13	-0.40	-0.18		-0.41
Sutterella sp..252	0.15			1.56		-3.33			1.96		0.32
Sutterella wadsworthensis		-0.12			0.63	0.48		-0.10		0.11	

Turicibacter sanguinis		-0.29								0.18	
Turicibacter sp..H121		0.50	-0.17	-0.29		-0.11					
Vallitalea pronyensis	0.27	0.68			0.62	-0.35	-0.14		0.15		-0.17
