# Supplementary material for: Associations Between the Gut Microbiota and Physical Activity, Sedentary Behaviour and Physical Function in Community‐Dwelling Older Adults
Source: J Aging Res. 2026 Apr 10;2026:8981398. doi: 10.1155/jare/8981398 (PMC13069175; doi:10.1155/jare/8981398)
Supplement: Supplementary file 1 — Supporting Information Additional supporting information can be found online in the Supporting Information section. [file JARE-2026-8981398-s001.zip › STROBE CHECKLIST.docx]

**STROBE Checklist for Cross-Sectional Studies**

| **Section** | **Item No.** | **STROBE Recommendation** | **Reported?** | **Page/Line Number** |
| --- | --- | --- | --- | --- |
| **Title and Abstract** | 1a | Indicate the study's design with a commonly used term in the title or the abstract | ✓ | Abstract, line 32 |
|  | 1b | Provide in the abstract an informative and balanced summary of what was done and what was found | ✓ | Abstract, lines 32-47 |
| **Introduction** |  |  |  |  |
| Background/Rationale | 2 | Explain the scientific background and rationale for the investigation being reported | ✓ | Pages 3-5 |
| Objectives | 3 | State specific objectives, including any prespecified hypotheses | ✓ | Page 5, line 99 |
| **Methods** |  |  |  |  |
| Study Design | 4 | Present key elements of study design early in the paper | ✓ | Page 6, line 115 |
| Setting | 5 | Describe the setting, locations, and relevant dates, including periods of recruitment, exposure, follow-up, and data collection | ✓ | Page 6 |
| Participants – Eligibility | 6a | Give the eligibility criteria, and the sources and methods of selection of participants | ✓ | Page 6 |
| Participants – Selection Method | 6b | Describe sources and methods of selection | ✓ | Page 6 |
| Variables – Definitions | 7 | Clearly define all outcomes, exposures, predictors, potential confounders, and effect modifiers. Give diagnostic criteria, if applicable | ✓ | Pages 7-11 |
| Data Sources – Measurement Methods | 8 | For each variable of interest, give sources of data and details of methods of assessment. Describe comparability of assessment methods if there is more than one group | ✓ | Pages 7-11 |
| Bias | 9 | Describe any efforts to address potential sources of bias | ✓ | Pages 6 and 9-11 |
| Study Size | 10 | Explain how the study size was arrived at | Partial. With n=101, a two-sided test (α=.05) provides 80% power to detect a correlation of approximately r=0.28 (and 90% power for r≈0.32), indicating the study is powered to detect moderate associations but may be underpowered for small effects. Given the large number of taxa tested and false discovery rate correction, power to detect small taxa-specific associations is likely reduced, and findings should be interpreted accordingly | Pages 6-7 |
| Quantitative Variables | 11 | Explain how quantitative variables were handled in the analyses. If applicable, describe which groupings were chosen and why | ✓ | Page 11 |
| Statistical Methods – Overall | 12a | Describe all statistical methods, including those used to control for confounding | ✓ | Page 11 |
| Statistical Methods – Subgroups | 12b | Describe any methods used to examine subgroups and interactions | N/A |  |
| Statistical Methods – Missing Data | 12c | Explain how missing data were addressed | There were no missing data for the primary variables, as all included participants had complete accelerometer and microbiome data. |  |
| Statistical Methods – Sampling Strategy | 12d | If applicable, describe analytical methods taking account of sampling strategy | N/A |  |
| Statistical Methods – Sensitivity Analysis | 12e | Describe any sensitivity analyses | N/A |  |
| **Results** |  |  |  |  |
| Participants – Flow | 13a | Report numbers of individuals at each stage of study (potentially eligible, examined for eligibility, confirmed eligible, included in study, completing follow-up, and analysed) | ✓ | Page 12 |
| Participants – Non-participation | 13b | Give reasons for non-participation at each stage | N/A |  |
| Participants – Flow Diagram | 13c | Consider use of a flow diagram | N/A |  |
| Descriptive Data – Characteristics | 14a | Give characteristics of study participants (demographic, clinical, social) and information on exposures and potential confounders | ✓ | Pages 12-13 |
| Descriptive Data – Missing Data | 14b | Indicate number of participants with missing data for each variable of interest | There were no missing data for the primary variables, as all included participants had complete accelerometer and microbiome data. |  |
| Outcome Data | 15 | Report numbers of outcome events or summary measures | ✓ | Pages 13-14 |
| Main Results – Unadjusted & Adjusted | 16a | Give unadjusted estimates and, if applicable, confounder-adjusted estimates and their precision (95% confidence interval). Make clear which confounders were adjusted for and why | ✓ | Pages 15-16 and 21-22 |
| Main Results – Category Boundaries | 16b | Report category boundaries when continuous variables were categorized | N/A |  |
| Main Results – Absolute Risk | 16c | If relevant, consider translating estimates of relative risk into absolute risk for a meaningful time period | N/A |  |
| Other Analyses – Subgroups & Interactions | 17 | Report other analyses done—eg analyses of subgroups and interactions, and sensitivity analyses | N/A |  |
| **Discussion** |  |  |  |  |
| Key Results | 18 | Summarise key results with reference to study objectives | ✓ | Page 25, line 434 |
| Limitations | 19 | Discuss limitations of the study, taking into account sources of potential bias or imprecision. Discuss both direction and magnitude of any potential bias | ✓ | Page 32, line 587 |
| Interpretation – Caution | 20 | Give a cautious overall interpretation of results considering objectives, limitations, multiplicity of analyses, results from similar studies, and other relevant evidence | ✓ | Page 32-33 |
| Generalisability | 21 | Discuss the generalisability (external validity) of the study results | ✓ | Page 32, line 590 |
| **Other Information** |  |  |  |  |
| Funding | 22 | Give the source of funding and the role of the funders for the present study and, if applicable, for the original study on which the present article is based | ✓ | Page 34, line 625 |
| Conflicts of Interest | 23 | Declare conflicts of interest of each author | ✓ | Page 34, line 622 |
