# Supplementary figures and images for: Associations Between the Gut Microbiota and Physical Activity, Sedentary Behaviour and Physical Function in Community‐Dwelling Older Adults
Source: J Aging Res. 2026 Apr 10;2026:8981398. doi: 10.1155/jare/8981398 (PMC13069175; doi:10.1155/jare/8981398)

## Slide 1
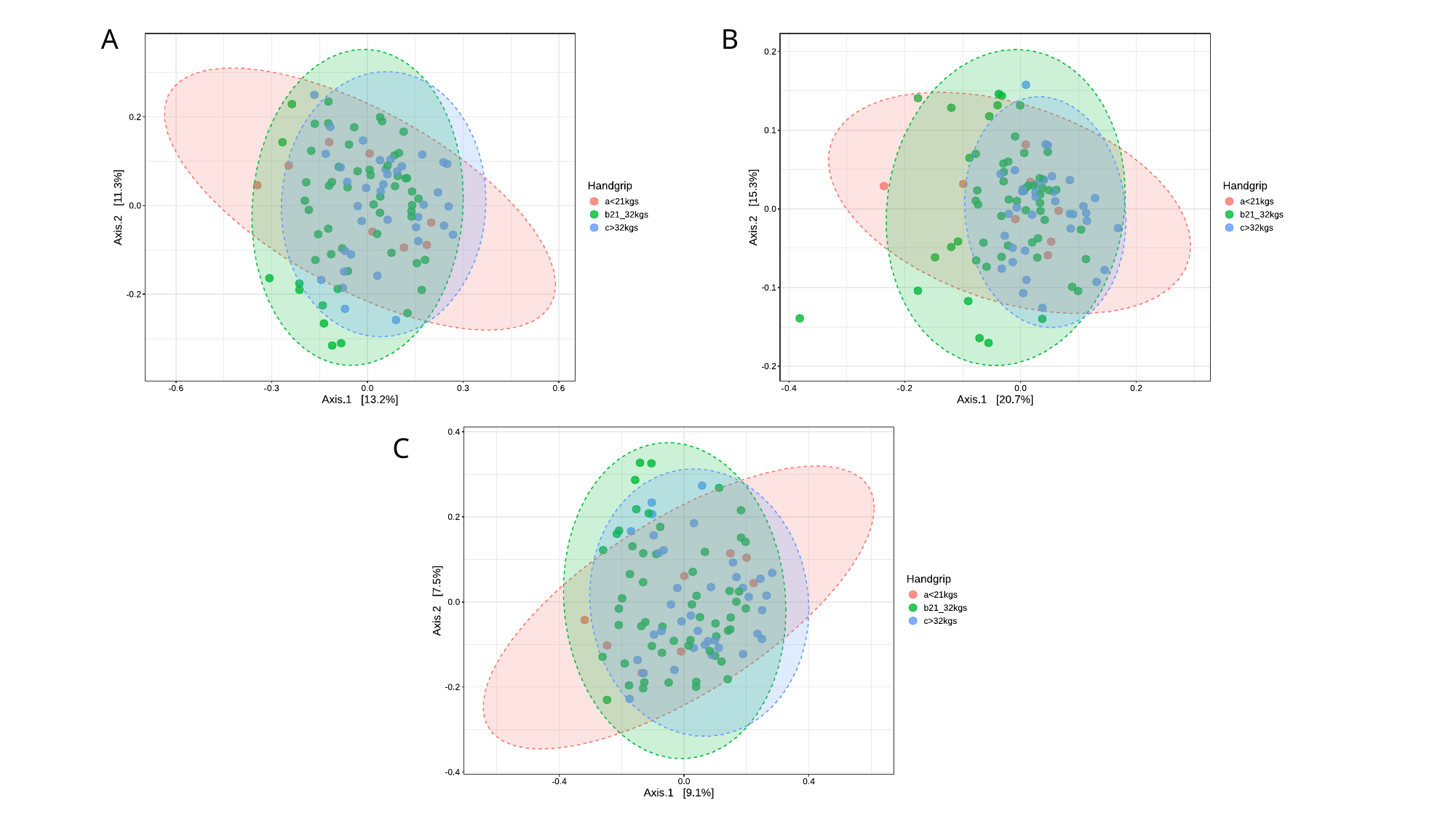

A
B
C

Supplement: Supplementary file 1 — Supporting Information Additional supporting information can be found online in the Supporting Information section. [file JARE-2026-8981398-s001.zip › Supplementary figure 2 - beta diversity indexes.pptx]
